# AGE-RELATED DIFFERENCES IN INHIBITION INVESTIGATION OF SIMON AND FLANKER CONFLICTS IN ERPS

**DOI:** 10.1093/geroni/igz038.2403

**Published:** 2019-11-08

**Authors:** Rachel Scrivano, Paul Kieffaber

**Affiliations:** William & Mary, Williamsburg, Virginia, United States

## Abstract

It is unclear whether or not older adults experience more difficulty managing cognitive conflict by inhibiting distracting stimuli and/or ignoring irrelevant information than younger adults. A common procedure used to measure inhibitory function is through the use of congruent and incongruent stimuli. Specifically, past literature that used tasks like the Simon and flanker have found differing effects on reaction times and various event-related potential (ERP) amplitudes and latencies, suggesting that either inhibitory function is a unitary mechanism or multifaceted. Moreover, research exhibits uncertainty for whether or not age influences deficits to inhibitory function. Therefore, the present study sought to investigate these research questions by combining the Simon and flanker tasks into one unique task. The study’s behavioral results indicate that older adults have greater difficulty with cognitive conflict in both Simon and flanker tasks due to significantly prolonged reaction times during incongruent trials. Furthermore, reaction time data posits that there are no significant age-related differences between the Simon and flanker task. This finding indicates that through the use of these tasks, inhibitory function is a unitary mechanism. In addition, preliminary electroencephalogram data shows that younger and older adults process the task’s information similarly. These initial findings can further inhibition research through its use as benchmarks for the measurement of cognitive change and deficit identification in older adults.

